# Potential effect of non-nitrogen fixing cyanobacteria *Spirulina platensis* on growth promotion of wheat (*Triticum aestivum* L.) under salt stress

**DOI:** 10.1038/s41598-025-14567-y

**Published:** 2025-08-08

**Authors:** Nabil Touzout, Mahfoud Ainas, Meriem Babaali, Hamza Moussa, Adil Mihoub, Iftikhar Ahmad, Aftab Jamal, Subhan Danish, Riaz Ahmad, Yaser Hassan Dewir, Árpád Székely

**Affiliations:** 1 Department of Agronomy, Faculty of Sciences, Pole Urban Ouzera, University of Medea, Medea, 26000 Algeria; 2 Laboratory Materials and Environment, University Yahia Fares of Medea, Urban Center, Medea, 26000 Algeria; 3 Laboratoire de Gestion et Valorisation des Ressources Naturelles et Assurance Qualité (LGVRNAQ), Faculté des Sciences de la Nature et de la Vie et des Sciences de la Terre, Université de Bouira, Bouira, 10000 Algeria; 4 Département des Sciences Biologiques, Faculté des Sciences de la Nature et de la Vie et des Sciences de la Terre, Université de Bouira, Bouira, 10000 Algeria; 5 Biophysical Environment Station, Center for Scientific and Technical Research on Arid Regions, Touggourt, Algeria; 6 Department of Environmental Sciences, COMSATS University Islamabad, Vehari Campus, Vehari, 61100 Pakistan; 7 Department of Soil and Environmental Sciences, Faculty of Crop Production Sciences, The University of Agriculture, Peshawar, 25130 Pakistan; 8 Pesticide Quality Control Laboratory, Agriculture Complex, Old Shujabad Road, Multan, 60000 Punjab Pakistan; 9 Department of Horticulture, The University of Agriculture, Dera Ismail Khan, 29220 Pakistan; 10 Department of Plant Production, College of Food and Agriculture Sciences, King Saud University, Riyadh, 11451 Saudi Arabia; 11 Research Centre for Irrigation and Water Management, Institute of Environmental Sciences, Hungarian University of Agriculture and Life Sciences, Anna-Liget str. 35, Szarvas, 5540 Hungary

**Keywords:** Biostimulation, Cyanobacteria, Oxidative stress, Crops tolerance, Soil salinization, Plant stress responses, Plant physiology

## Abstract

Numerous microalgae have been used as modern eco-friendly biostimulants under abiotic stress conditions; however, the application of non-nitrogen fixing cyanobacteria, such as *Spirulina (Arthrospira platensis)* has not been extensively investigated. In this study, the effects of *A. platensis* (60 mg/L) applied twice as a foliar application on the growth, photosynthetic pigments, and oxidative metabolism of *Triticum aestivum* seedlings grown under salt stress (150 mM) were evaluated. Under salt stress conditions, growth attributes such as shoot and roots fresh weights, lengths, and photosynthetic pigments were significantly inhibited compared to the control group. Treatment with *A. platensis* effectively improved all growth parameters. Under salt stress conditions, shoot fresh weight and length increased by 49% and 44%, respectively, while root fresh weight and length were enhanced by 105% and 223%. The contents of chlorophyll a, b, and carotenoids in wheat were significantly reduced by 57%, 35%, and 43%, respectively. Additionally, seedlings exposed to salinity showed improved accumulation of hydrogen peroxide (H_2_O_2_) and malondialdehyde (MDA), along with decreased peroxidase (POD) enzyme activity. *Spirulina* extract (SPE) mitigated salt and induced oxidative stress by enhancing the activities of antioxidant enzymes. Furthermore, SPE protected wheat seedlings from the detrimental effects of H_2_O_2_ by promoting secondary metabolite biosynthesis. Additionally, SPE increased the proline content by 25%, aiding in the regulation of osmotic stress. Taken together, the results of this study support the application of *A. platensis* as an effective biostimulant for improving wheat growth and food security by reducing the harmful impacts of salt stress in semi-arid regions.

## Introduction

Salt-affected soil is a significant abiotic stress factor that adversely affects crop growth and production worldwide, posing a serious challenge to sustainable agriculture, particularly in arid and semi-arid regions^1^. Salt stress disrupts plant nutrient balance, water uptake, and metabolic processes, leading to ionic, osmotic, and oxidative stress, ultimately resulting in lower crop yields^2^. To mitigate these harmful effects, biostimulants have emerged as an effective approach for enhancing plant resilience to salinity stress without relying on chemical fertilizers. Among various natural biostimulants, microalgae have gained attention for their potential to alleviate the injurious effects of salt stress in crops^3–5^.

The application of microalgae as a biostimulant in modern agriculture presents a sustainable and eco-friendly approach to improving plant resilience to abiotic stress, potentially increasing productivity in salt-affected soils. By improving plant growth and productivity under salt stress, microalgae contribute to both sustainable agriculture and environmental protection, reducing the reliance on synthetic fertilizers^6^. *Arthrospira platensis*, a blue-green cyanobacteria, is rich in bioactive compounds, including proteins, essential fatty acids, minerals, phytohormones, and vitamins, which play a vital role in improving plant tolerance to abiotic stresses^7,8^. As a biostimulant, *A. platensis* can improve nutrient absorption, root growth, and the antioxidant defense system, helping plants adapt to salt stress more effectively. The accumulation of antioxidants in *A. platensis*, such as phycocyanin, helps neutralize reactive oxygen species (ROS) that are overproduced under saline conditions, alleviating oxidative stress and supporting plant growth^9^. Moreover, *A. platensis* extracts have been found to contain natural phytohormones like cytokinins and auxins, which modulate growth and stress tolerance in crops, promoting cell division and elongation even under salt stress^10,11^.

Although recent studies suggest that *spirulina* extract can alleviate abiotic stress-induced phytotoxicity by modulating redox homeostasis and secondary metabolite biosynthesis^9–14^, the precise underlying mechanisms remain insufficiently understood. Given its bioactive properties, this study hypothesized that exogenous application of *Spirulina platensis* extract (SPE) would enhance NaCl tolerance in wheat plants by strengthening the antioxidant defense system. This study aimed to explain the physiological and biochemical mechanisms through which SPE mitigated salt stress, providing insights into its potential role as a natural biostimulant for improving crop resilience in saline environments. Additionally, we evaluated the impact of SPE root application on growth attributes, photosynthetic pigments, oxidative stress markers, and the antioxidant defense system under NaCl stress. Overall, this study offers a theoretical framework for utilizing SPE as a biofertilizer to mitigate the detrimental effects of saline soil in agricultural crops.

## Materials and methods

### *Spirulina platensis* biomass Preparation

The *Spirulina platenis* strain used in this study originated from Tamanrasset (southern Algeria) and was provided to the Algiers Biotechnology and Process Engineering Laboratory, Department of Environment, Algiers Polytechnic School, Algeria. To confirm the strain’s authenticity, its morphological characteristics were examined under a light microscope, assessing its filamentous structure, spiral shape, and cellular arrangement. Additionally, physiological traits such as growth rate and pigment composition were analyzed to ensure consistency with known *A. platensis* profiles^15^. The *Spirulina* strain was chosen based on its ability to quickly accumulate biomass and its well-known richness in biofertilizer. The *A. platensis* strain was cultivated on the standard Zarrouk culture medium^16^ in cylindrical flasks (500 mL capacity) with an initial volume of 250 mL. The flasks were incubated in an algal growth chamber under controlled conditions: aerated with air pumps with a flow rate of 60 L/h of filtered air and incubated at 30 ± 2 °C with fluorescent lamps (Torch^®^) emitted light white intensity of 36 µmol m^−2^s^−1^. The pH was maintained at 9 ± 0.2. To sustain the culture in the exponential phase, fresh Zarrouk medium was added every 7 days (dilution in half). A portion of the culture was used for the experiment, while the remainder was preserved for future use.

From a mature culture in a 100 mL Erlenmeyer flask, the biomass was diluted to a final concentration of approximately 1 g/L of fresh algal biomass. Before applying live cell treatment, the biomass was centrifuged at 4500 rpm for 15 min. The cell pellets were washed twice and resuspended with sterile physiological H_2_O to remove Zarrouk traces. The dry weight of the algal biomass was estimated indirectly by measuring the optical density (OD) of the suspension at 618 nm using a spectrophotometer. A calibration curve was established to relate OD values to dry weight concentration, following the method described by Touzout et al.^17^. The relationship between OD and dry weight was expressed using the following equation:

$$\:OD=1.1482\:C+0.085$$

OD is the optical density of the suspension,

### Preparation of *A. platensis* suspension

The cyanobacterial samples were harvested at the end of the stationary growth phase through centrifugation. The resulting pellets were collected, evenly spread in a thin layer on glass plates, and air-dried under a mild airflow. The air-dried biomass was then transferred to a porcelain crucible and further dried at 50 °C until a constant weight was achieved. A total of 5 g of the dried biomass was suspended in 50 mL of distilled water to prepare the foliar spray solution for the treated potted plants.

### Soil, plant material and growth conditions

This work was carried out in the Laboratory of Microbiology at Médéa. Soil sampling was conducted from a homogenized plot at a depth of 15 to 20 cm in the region of Gtitene town, Médéa, Algeria. The collected soil samples were air-dried overnight and then passed through a 2 mm sieve. The soil was then transferred to plastic pots with the following dimensions: 40 × 30 cm, containing 1 kg of soil per pot. To characterize the baseline soil conditions, both electrical conductivity (EC) and pH were measured before and after salt treatment. Prior to treatment, the soil had an EC of 1.2 dS m^−1^ and a pH of 7.4, indicating non-saline, near-neutral conditions. Following the addition of 150 mM NaCl, the EC increased to 5.8 dS m^−1^ and the pH slightly decreased to 7.2, confirming the induction of salinity stress.

Wheat (var. SEMITO) seeds were obtained from the Institute Technique des Grandes Cultures (ITGC) de Berouaghia (Médéa, Algeria). The seeds were surface sterilized with 1% sodium hypochlorite for 30 min, thoroughly washed with double deionized water and soaked for 72 h. Seeds were sown in plastic pots containing 1 kg of soil and grown at a density of five seeds per pot after germination. The pots were maintained under controlled greenhouse conditions for 90 days (December to February), with minimum and maximum average temperatures of 18–26 °C, a 14/8 hr day/night photoperiod, and manual irrigation up to 90% of the soil holding capacity, allowing leaching during the treatment period.

The experiment followed a completely Randomized Design with four treatments, each replicated 4 times: CK (control), NaCl (150 mM NaCl), SPE (60 mg/L, *A. platensis*), and NaCl-SPE (150 mM NaCl + 60 mg/L *A. platensis*). The experimental conditions were identical to those used during seed treatment. Foliar spraying was initiated 40 days after germination, corresponding to Feekes growth stage 6 (stem elongation stage). Each plant received foliar spraying (60 mg/L per plant) every 5 days for a period of 20 days using a dorsal sprayer (1 L). The selected concentration of 60 mg/L for *A. platensis* extract was based on preliminary in-house experiments which indicated this dose provided optimal effects without signs of phytotoxicity and prior studies^13,18–20^. Before spraying, the soil surface in each pot was completely covered with aluminum foil to prevent leaching of the spray into the soil, which could lead to uptake by the roots. Foliar application of cyanobacterial cellular extracts was conducted during the noon time, when the plant received the maximum possible sunlight, resulting in wider stomatal openings. The increased water pressure during noon facilitated greater penetration of the extracts into the leaves through the stomata.

#### Growth attributes

After six weeks of treatment, plants from each group were carefully uprooted without damaging the roots, washed with distilled water to remove soil particles, and gently dried by tapping between filter paper. The fresh biomass was then measured using a digital electronic scale. The length of the aerial and root parts was recorded using a graduated ruler.

#### Determination of photosynthetic pigments

The content of pigments was assessed according to the method described by Lichtenthaler^21^.

#### Determination of oxidative stress biomarkers

A sample of 0.5 g of weighed and ground leaves was homogenized in 0.1% (w/v) trichloroacetic acid (TCA) and centrifuged at 15,000 g for 12 min at 4 °C. The supernatant was used for the determination of hydrogen peroxide (H_2_O_2_) and malonaldehyde (MDA) contents. As outlined by Velikova et al.^22^, the amount of H_2_O_2_ formed in H_2_O_2_-KI reaction was determined. Lipid peroxidation was assessed by measuring the levels of malondialdehyde (MDA) as proposed by Heath and Packer^23^. Spectrophotometric measurement of MDA was performed at wavelengths of 530 and 600 nm. The results were expressed in the final data in µmol g^−1^ FW.

#### Determination of antioxidant enzymes

Ascorbate peroxidase (APX) activity was assessed as described by Nakano and Asada^24^ by monitoring the reduction in ascorbic acid absorbance at 290 nm. Peroxidase (POD) activity was measured by the method described by Cakmak and Marschner^25^. The calculation of phenylalanine ammonia-lyase (PAL) was evaluated by spectrophotometric methods measuring the conversion of phenylalanine to transcinnamic acid monitored at a wavelength of about 290 nm^26^.

#### Determination of non-enzymatic antioxidants

The ninhydrin method described by Bates et al.^27^ was applied in measurement of leaves proline content. Total phenolic contents were calculated following the colorimetric method of Folin-Ciocalteu as described by Ainsworth et al.^28^. Gallic acid was used as a reference when measuring absorbance at 760 nm. The content of flavonoids compounds was determined according to Zhishen et al.^29^ procedures, using quercetin as a reference. The recycling method of Anderson^30^, was used to assess leaves GSH levels.

### Statistical analysis

Data are presented as means ± standard deviation (SD) and were subjected to one-way analysis of variance (ANOVA), followed by HSD Tukey test at *P <* 0.05 using IBM SPSS statistical software (ver.22.0, SPSS Inc., Chicago, IL, USA). Before conducting ANOVA and correlation analyses, data distribution was assessed using the Shapiro–Wilk normality test. The majority of physiological and biochemical parameters showed no normal distribution (*p* > 0.05). Therefore, Spearman’s rank correlation was also computed to confirm the reliability of the observed associations. Additionally, principal component analysis (PCA), hierarchical clustering, and heatmaps were generated using Origin software (OriginLab, 2021) to explore multivariate patterns among treatments and indicators.

## Results

### SPE amendment enhances wheat growth attributes under salt stress

**Fig. 1:**
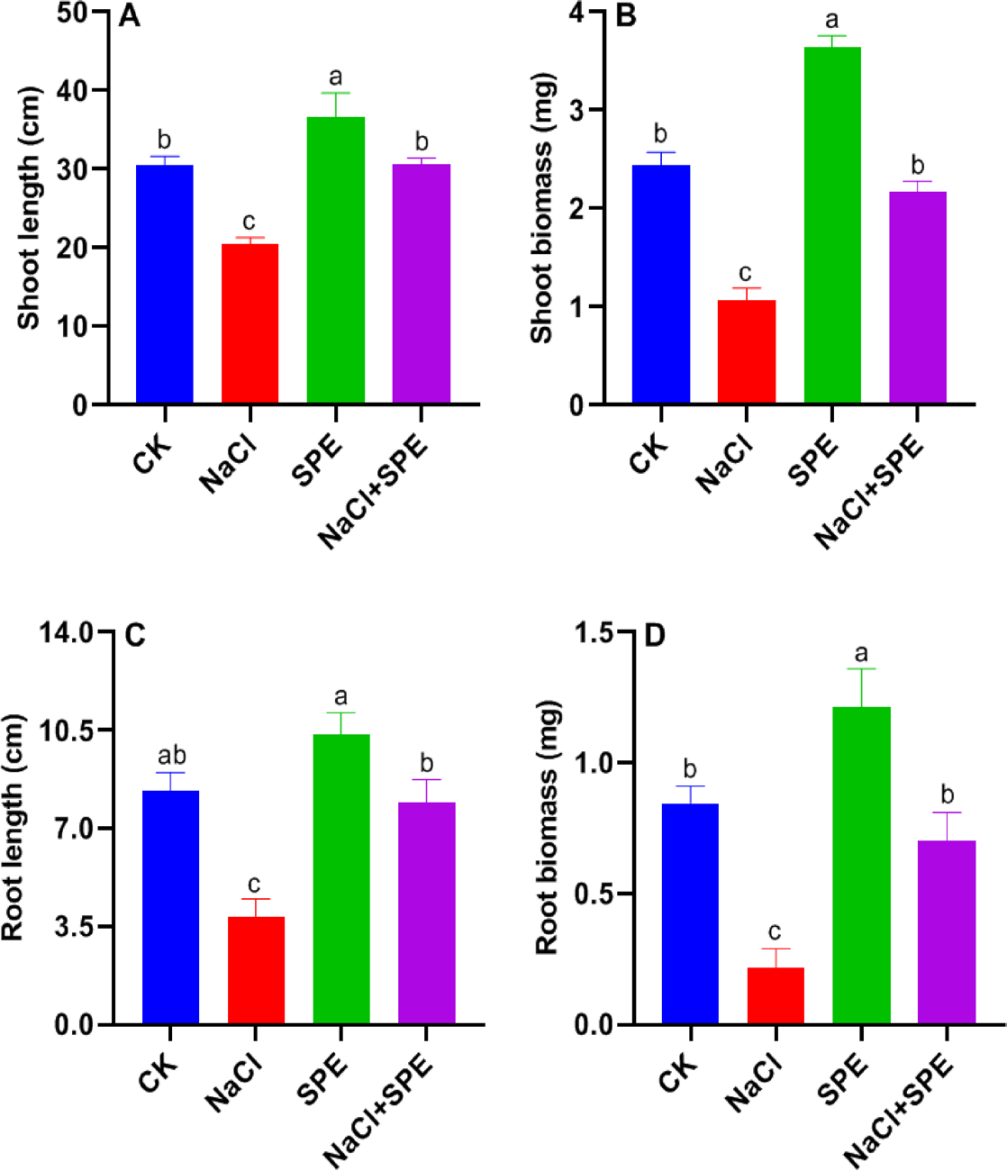
Effect of *Spirulina* Extract (SPE) application on NaCl-induced growth inhibition in wheat seedlings. (**A**) Shoot length, (**B**) Shoot fresh biomass, (**C**) Root length, and (**D**) Root fresh biomass. Treatment groups include CK (Control) and SPE (*Spirulina* Extract) under salt stress (NaCl). Results are presented as mean ± SD (*n* = 4 replicates), with different letters indicating significant differences according to Tukey’s test at *P* < 0.05.

The results on wheat plants growth attributes, including fresh weight and shoot/root length, varied in response to different treatments, as shown in Fig. 1. Salt stress significantly reduced shoot and root fresh weight by 56.47 and 74.34%, respectively, compared to the control group. Additionally, salt stress decreased shoot and root length by 32.79% and 53.9%, respectively, relative to the control group. Interestingly, *Spirulina* extract (SPE) alone and in combination with NaCl treatments significantly improved all growth attributes (fresh weight and shoot/root length) compared to both the NaCl treatment and the control group (Fig. 1). The SPE treatment notably increased shoot fresh weight and length by 49.19 and 43.76%, respectively, compared to the control group. Moreover, applying *Spirulina* extract (SPE) to NaCl treated plants significantly increased root fresh weight and length by 223.50% and 104.19, respectively, compared to the NaCl group (Fig. 1B and D). Shoot and root length followed a similar trend in response to NaCl and SPE treatments (Fig. 1A and C). Overall, SPE amendment alleviates the negative effects of salinity and significantly enhances all growth parameters in wheat plants.

### SPE amendment enhances wheat photosynthetic pigments under salt stress

**Fig. 2:**
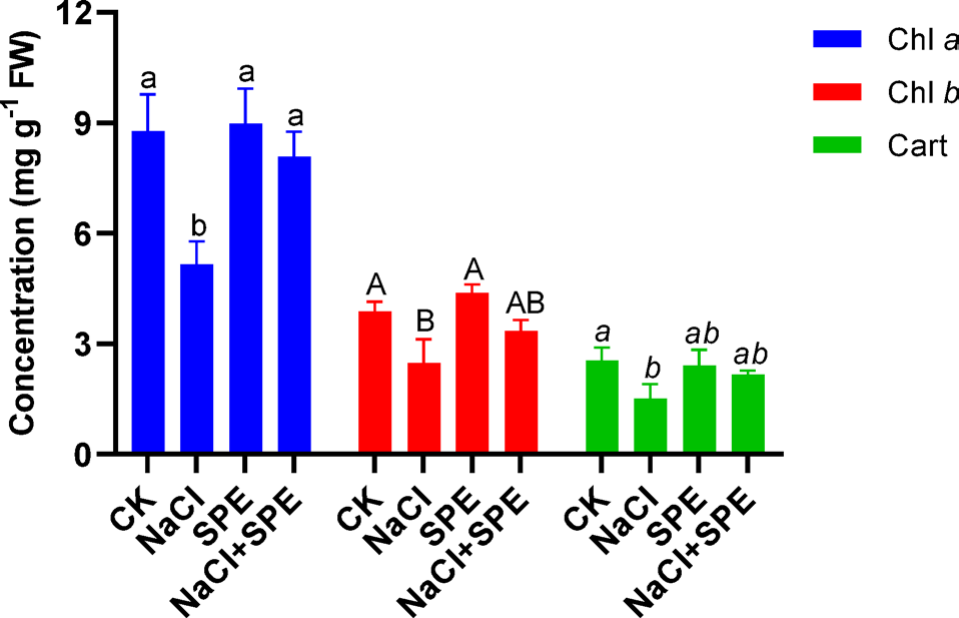
Effect of *Spirulina* Extract (SPE) application on NaCl-induced inhibition of pigment biosynthesis in wheat seedlings, including chlorophyll a (Chl a), chlorophyll b (Chl b), and carotenoid (Cart) content. Treatment groups include CK (Control) and SPE (*Spirulina* Extract) under salt stress (NaCl). Results are presented as mean ± SD (*n* = 4 replicates), with different letters indicating significant differences according to Tukey’s test at *P* < 0.05.

Salt stress significantly reduced the content of chlorophyll a, b and carotenoids by 41.25, 36.2, 40.29% respectively compared to the control plant (Fig. 2). However, the application of SPE resulted in a significant increase in pigment content compared to the control group (Fig. 2). Interestingly, the exogenous application of SPE significantly improved the content of chlorophyll a, b and carotenoids by in salt-stressed plants (Fig. 2). Specifically, SPE treatment increased chlorophyll a by 56.65%, chlorophyll-b by 35.34% and carotenoids by 42.85% in the presence of salt compared to salt stressed plants (Fig. 2). Overall, the application of SPE to the leaves enhanced the photosynthetic pigment content of wheat plants under salt stress.

### SPE amendment alleviates oxidative stress in wheat under salt stress

**Fig. 3:**
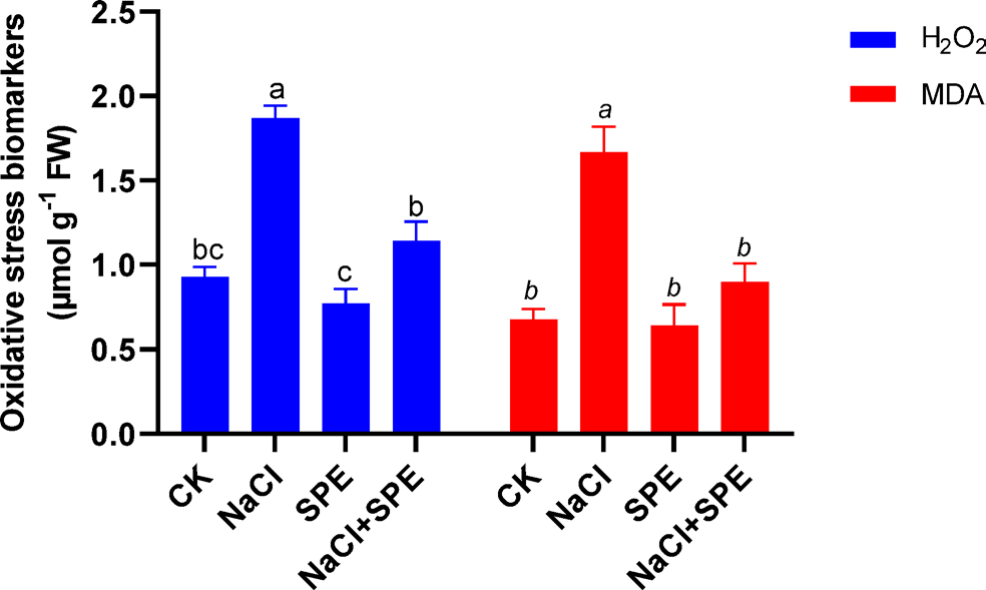
Effect of *Spirulina* Extract (SPE) application on NaCl-induced oxidative damage in wheat seedlings, represented by hydrogen peroxide (H₂O₂), and malondialdehyde (MDA) content. Treatment groups include CK (Control) and SPE (*Spirulina* Extract) under salt stress (NaCl). Results are presented as mean ± SD (*n* = 4 replicates), with different letters indicating significant differences according to Tukey’s test at *P* < 0.05.

Salt exposure induced oxidative stress in wheat plants, as evidenced by increased accumulation of H_2_O_2_ and MDA (Fig. 3). Salt treatment enhanced H_2_O_2_ and MDA content by 72.94% and 146.19%, respectively, in wheat leaves compared to control group (Fig. 3). However, SPE amendment effectively reduced H_2_O_2_ and MDA accumulation by 38.97% and 46.23%, respectively, in wheat plants under NaCl stress compared to NaCl stressed plants without SPE application (Fig. 3). Overall, our data demonstrates that SPE amendment can potentially alleviate NaCl induced phytotoxicity and promote wheat plants growth by mitigating lipid membrane peroxidation.

### SPE amendment enhances antioxidant enzymes activity in wheat under salt stress

**Fig. 4:**
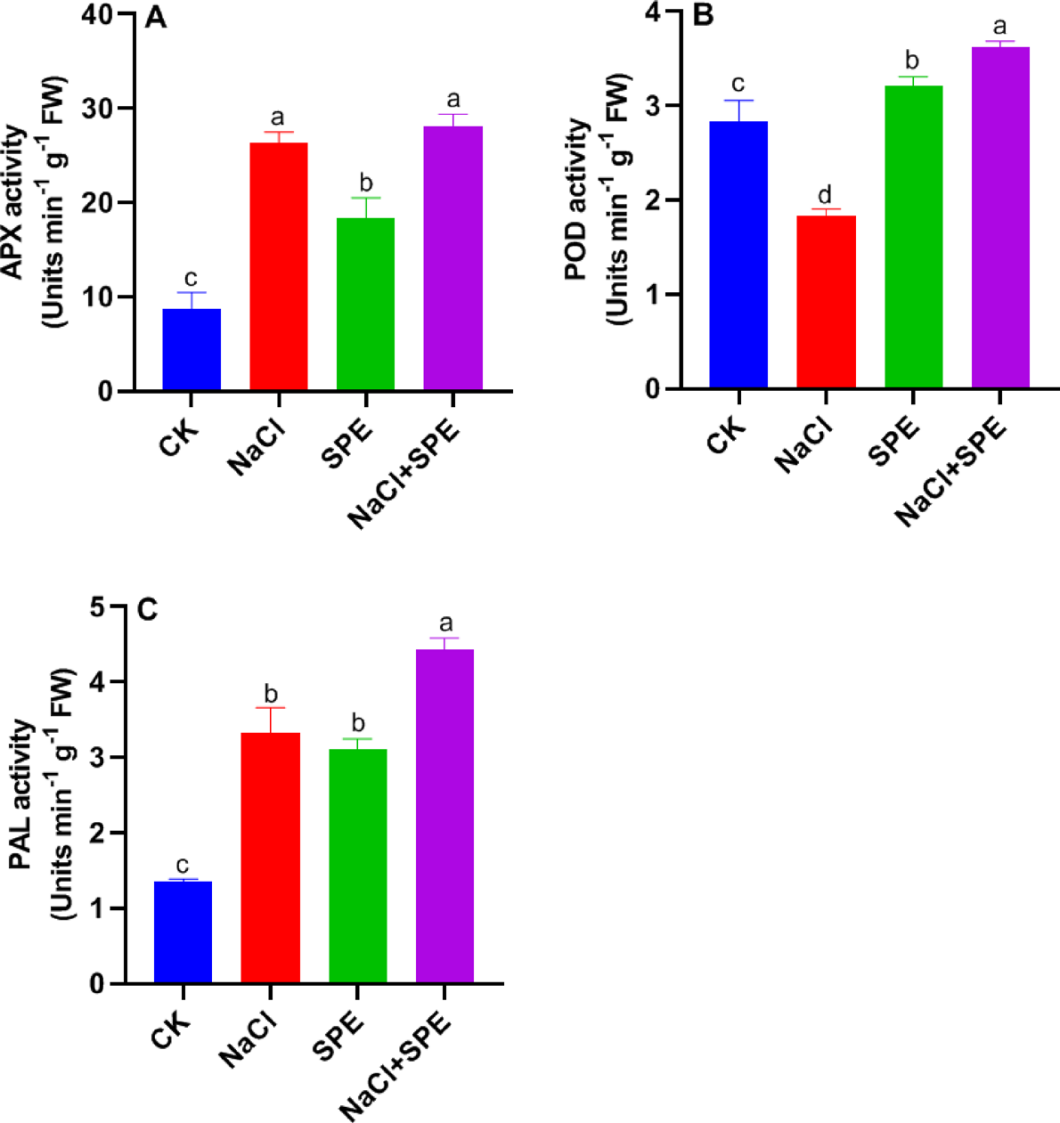
Effect of *Spirulina* Extract (SPE) application on NaCl-induced alterations in antioxidant enzyme activities in wheat seedlings. (**A**) Ascorbate peroxidase (APX), (**B**) Peroxidase (POX), and (**C**) Phenylalanine ammonia-lyase (PAL) activities. Treatment groups include CK (Control) and SPE (*Spirulina* Extract) under salt stress (NaCl). Results are presented as mean ± SD (*n* = 4 replicates), with different letters indicating significant differences according to Tukey’s test at *P* < 0.05.

The activity of the APX enzyme increased significantly by 202.49% in wheat leaves under salt stress compared to control group (Fig. 4A). In SPE-treated wheat seedlings, APX activity also increased by 110.69% compared to the control. Moreover, in seedlings treated with both SPE and salt, the increase was further elevated to 224.48% (Fig. 4A), suggesting that the mitigatory role of SPE in oxidative stress may depend on APX activity. Salt stress significantly reduced POD activity by 35.26% relative to the untreated control (Fig. 4B). However, POD activity in the leaves of SPE treated wheat seedlings was significantly enhanced (Fig. 4B). Compared to NaCl treatment alone, the application of SPE increased POD activity by 97.53% (Fig. 4B). The PAL enzyme activity in the leaves of salt treated wheat seedlings increased significantly by 146.80% compared to the control group (Fig. 4C). Interestingly, PAL activity was also up regulated by SPE treatment alone, and this induction was further enhanced following the co-application of NaCl and SPE (Fig. 4C), suggesting that the alleviatory role of SPE in salt stress may be dependent on the stimulation of the phenylpropanoid pathway.

### SPE amendment improves antioxidant metabolites contents in wheat under salt stress

**Fig. 5:**
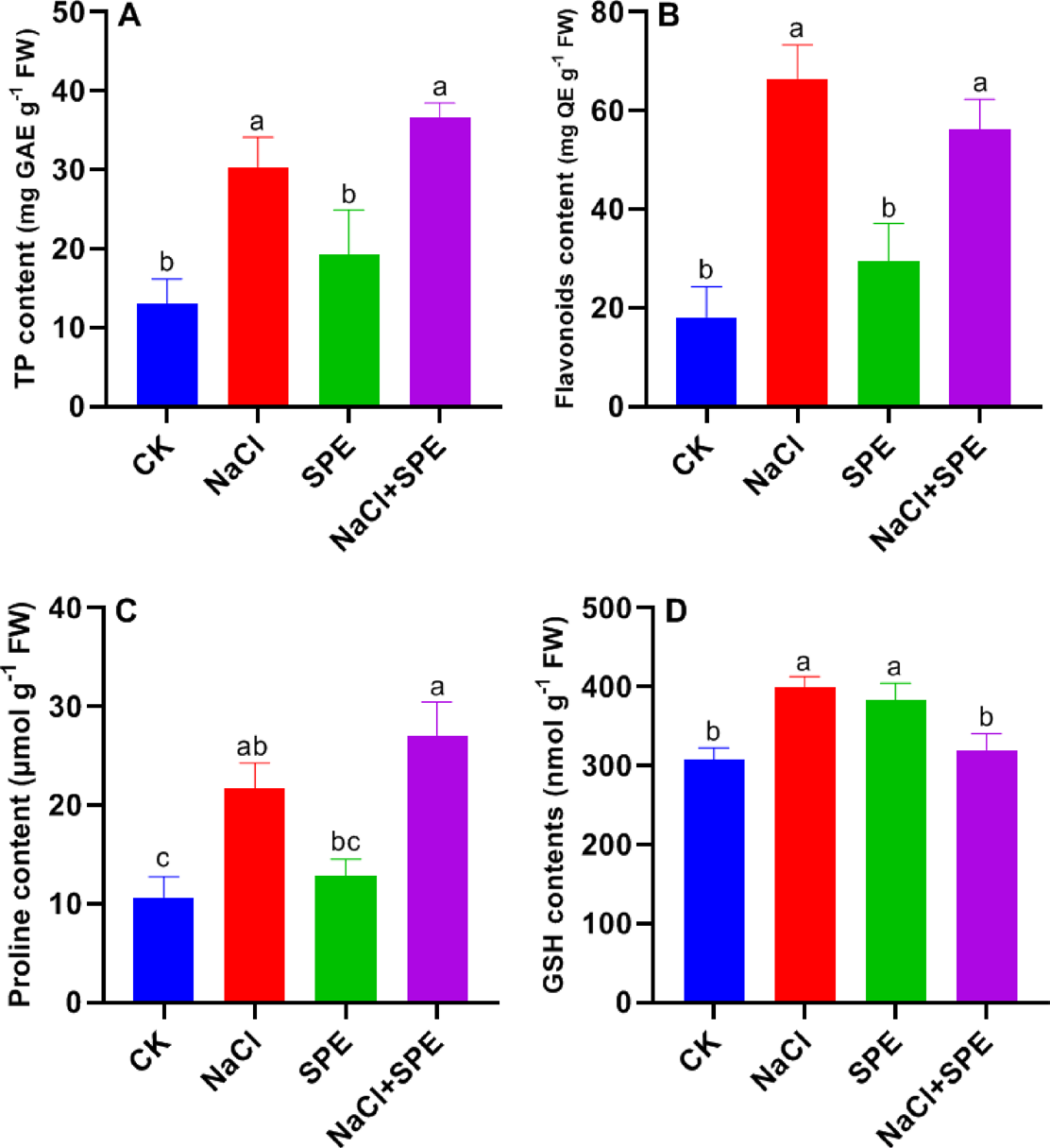
Effect of *Spirulina* Extract (SPE) application on NaCl-induced stimulation of antioxidant metabolites in wheat seedlings. (**A**) Total phenolics, (**B**) Flavonoids, (**C**) Proline, and (**D**) Reduced glutathione (GSH) content. Treatment groups include CK (Control) and SPE (*Spirulina* Extract) under salt stress (NaCl). Results are presented as mean ± SD (*n* = 4 replicates), with different letters indicating significant differences according to Tukey’s test at *P* < 0.05.

Salt application significantly increased total phenolic and flavonoids contents in wheat seedling leaves by 131.98% and 267.74% respectively, compared to the control group (Fig. 5A and B). SPE application further enhanced the accumulation of secondary metabolites compared to the untreated control (Fig. 5A and B). Specifically, EPS application increased total phenolic content by 20.76% (Fig. 4A), while reducing flavonoids levels by 15.54% (Fig. 5B) compared to salt treatment alone.

Wheat seedlings exhibited a significant increase in proline content under NaCl stress compared to the control group. Exogenous SPE applications further increased proline accumulation under salt stress. Proline levels were assessed in both stressed and control conditions following SPE treatment. Additionally, the content of low molecular weight thiols (GSH) increased by 29.63% under NaCl stress compared to control seedlings (Fig. 5D). Interestingly, exogenous SPE application significantly increased GSH content. However, compared to NaCl-stressed seedlings, SPE treatment led to a 19.73% reduction in GSH levels (Fig. 5D).

### Principal component analysis (PCA)

**Fig. 6:**
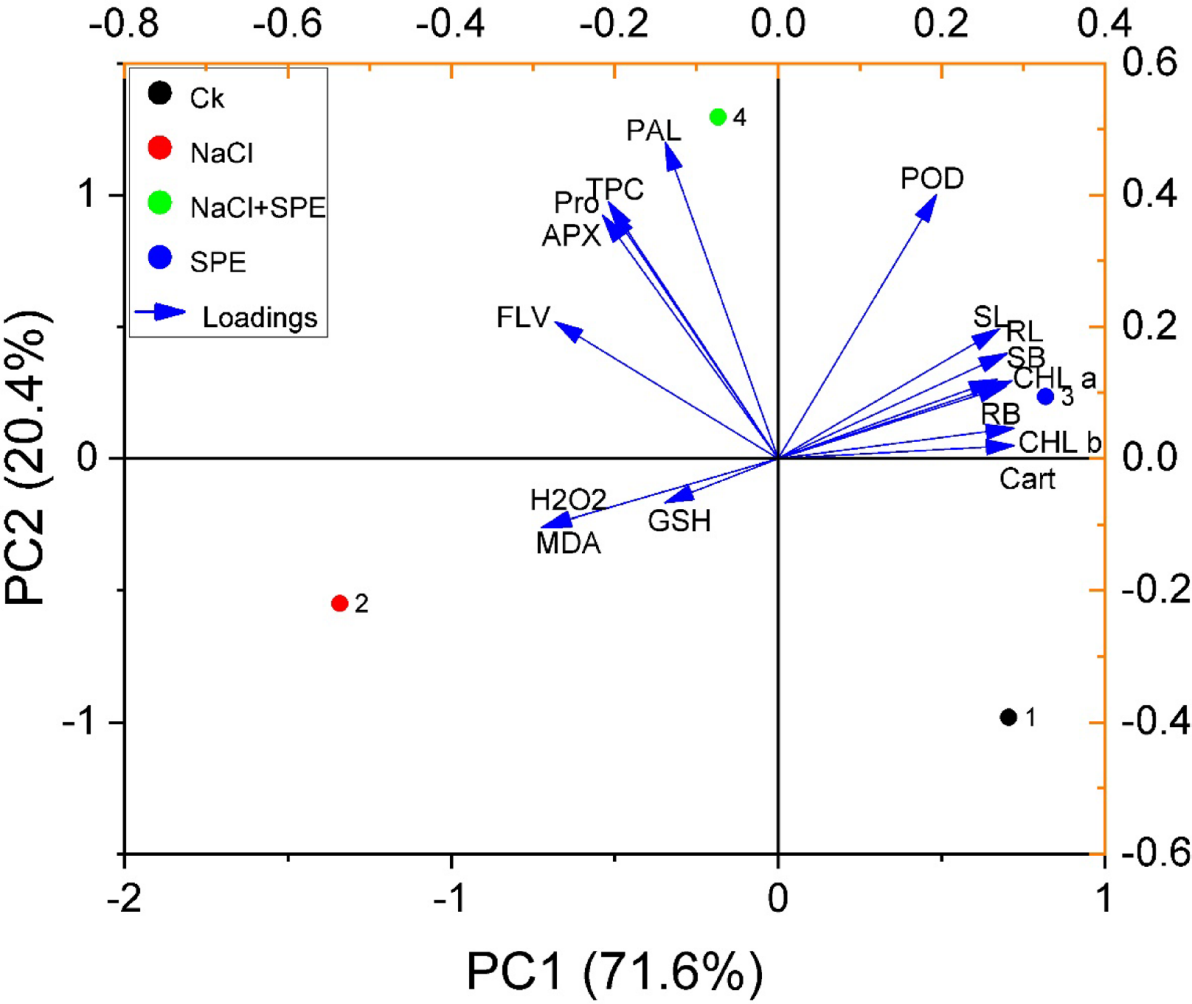
Principal component analysis (PCA) biplot of the studied traits and treatment combinations. The 95% confidence ellipse indicates treatment variability, with outliers falling outside the ellipse. (SL) shoot length, (SB) shoot fresh biomass, (RL) root length, (RB) root fresh biomass, (Chl a) chlorophyll a, (Chl b) chlorophyll b, (Cart) carotenoid contents, (H_2_O_2_) hydrogen peroxide, (MDA) malondialdehyde, (APX) ascorbate peroxidase, (POX) peroxidase, (PAL) phenylalanine ammonia-lyase activity, (TPC) total phenolic, (FLAV) flavonoids, (Pro) Proline, (GSH) reduced glutathione content. Treatment groups include CK (Control) and SPE (Spirulina Extract) under salt stress (NaCl).

The PCA biplot (Fig. 6) and eigenvalues from Table 1 illustrate the contributions of various traits (dependent variables) and treatments (independent variables), with PC1 explaining 71.6% and PC2 accounting for 20.4% of the total variance, collectively capturing 91.9% of the dataset’s variability. Table 1 highlights that traits such as SL, RL, SB, RB, CHL a, CHL b, CAR, H_2_O_2_, MDA, APX, FLV and Pro contribute the most to PC1, indicating their dominant role in explaining overall variance. The PCA-based biplot using these components demonstrates associations between morphological traits (SL, RL, SB, RB, CHL a, CHL b, and CAR) and the SPE treatment, indicating improved plant performance and quality under this condition. This reflects the positive effects of SPE on wheat growth promotion and related parameters under salt stress. Compounds such as H_2_O_2_, MDA and GST are more closely associated with the physiological responses of plants subjected to NaCl treatments, highlighting the complex interplay between salinity stress and plant defense mechanisms. Parameters including PAL, TPC, Pro, APX, FLV, and POD are more strongly linked to the combined SPE + NaCl treatment. Conversely, control treatments show negative correlations with all measured parameters.

**Table 1:** Summary of the principal component analysis of the measured trails (Squared cosines of the variables).

Variables	PC1	PC2
SB	**0.8223**	0.0469
SL	**0.8412**	0.1263
RB	**0.8941**	0.0395
RL	**0.8998**	0.0834
CHL a	**0.9404**	0.0455
CHL b	**0.9564**	0.007
Cart	**0.957**	0.0013
H_2_O_2_	**0.9642**	0.0357
MDA	**0.9496**	0.035
APX	**0.5278**	0.4449
POD	0.4293	**0.5237**
PAL	0.2173	**0.7511**
TPC	0.495	**0.4958**
FLV	**0.8544**	0.1406
Pro	**0.4837**	0.4684
GSH	0.2205	0.0147
**Eigen-value**	**11.45306**	**3.25971**
**Variance (%)**	**71.6**	**20.4**
**Cumulative (%)**	**71.6**	**91.95**

PC1 and PC2 are the first two components (PCs) with eigenvalues ≥ 1. Values in bold correspond for each variable to the factor for which the squared cosine is the largest. (SL) shoot length, (SB) shoot fresh biomass, (RL) root length, (RB) root fresh biomass, (Chl a) chlorophyll a, (Chl b) chlorophyll b, (Cart) carotenoid contents, (H2O2) hydrogen peroxide, (MDA) malondialdehyde, (APX) ascorbate peroxidase, (POX) peroxidase, (PAL) phenylalanine ammonia-lyase activity, (TPC) total phenolic, (FLAV) flavonoids, (Pro) Proline, (GSH) reduced glutathione content.

### Hierarchical cluster analysis

**Fig. 7:**
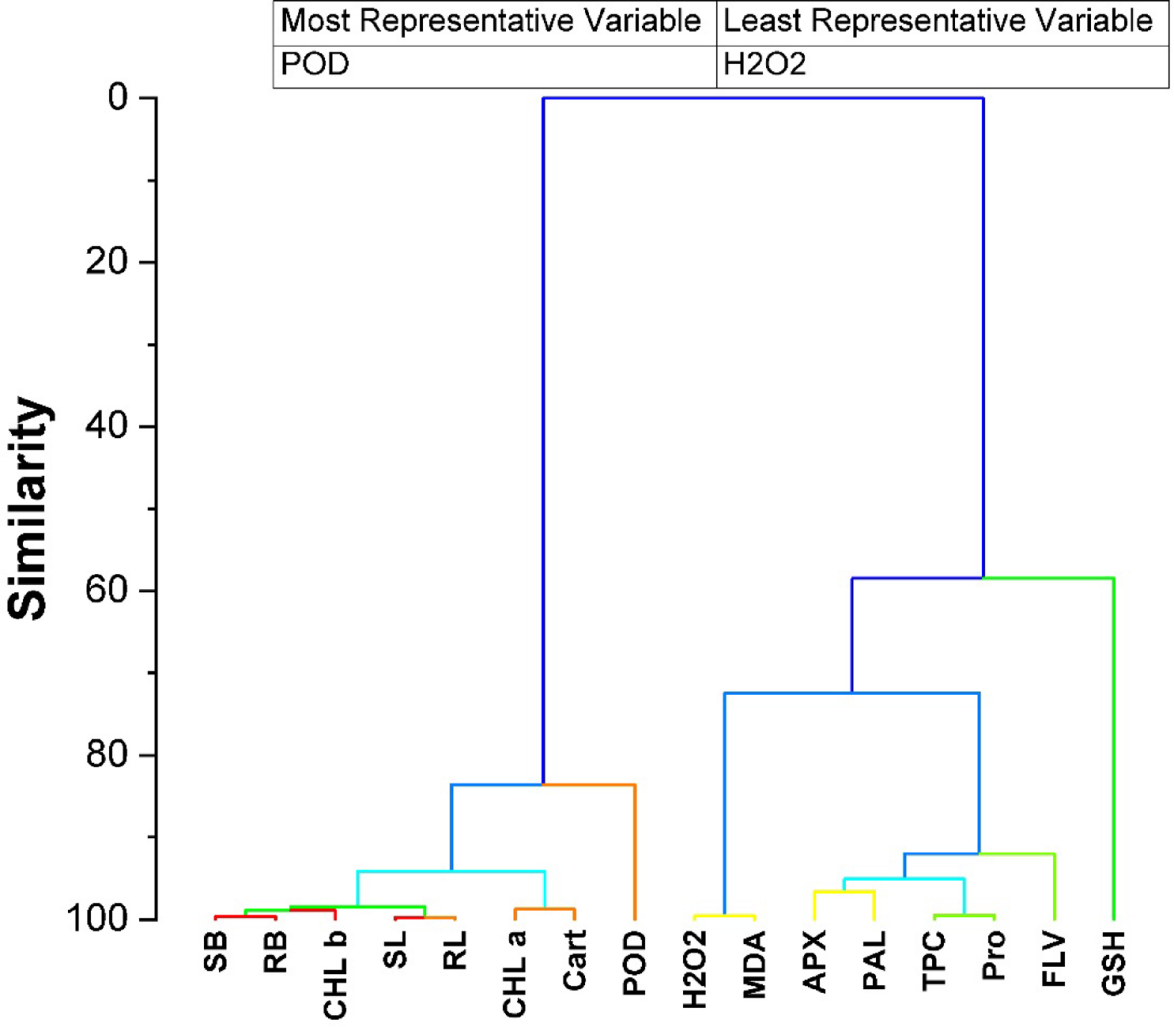
Hierarchical cluster plot for studied attributes. (SL) shoot length, (SB) shoot fresh biomass, (RL) root length, (RB) root fresh biomass, (Chl a) chlorophyll a, (Chl b) chlorophyll b, (Cart) carotenoid contents, (H_2_O_2_) hydrogen peroxide, (MDA) malondialdehyde, (APX) ascorbate peroxidase, (POX) peroxidase, (PAL) phenylalanine ammonia-lyase activity, (TPC) total phenolic, (FLV) flavonoids, (Pro) Proline, (GSH) reduced glutathione content.

The hierarchical cluster plot demonstrates that photosynthetic pigments and plant growth parameters are clustered together, indicating that optimal plant development is closely linked to high pigment production. Additionally, Fig. 7 highlighted that peroxidase (POD) was the most indicative variable, while hydrogen peroxide (H_2_O_2_) was the representative variable. This analysis offers valuable insights into the complex interactions among treatments, salt stress, and plant attributes.

### Spearman correlation analysis

From Spearman correlation analysis, a strong positive correlation exists between shoot and root length. This indicates that as plant height increases, root length also grows. The significant correlation between shoot biomass and root biomass suggests that an increase in shoot fresh weight leads to an improvement in root fresh weight.

**Fig. 8:**
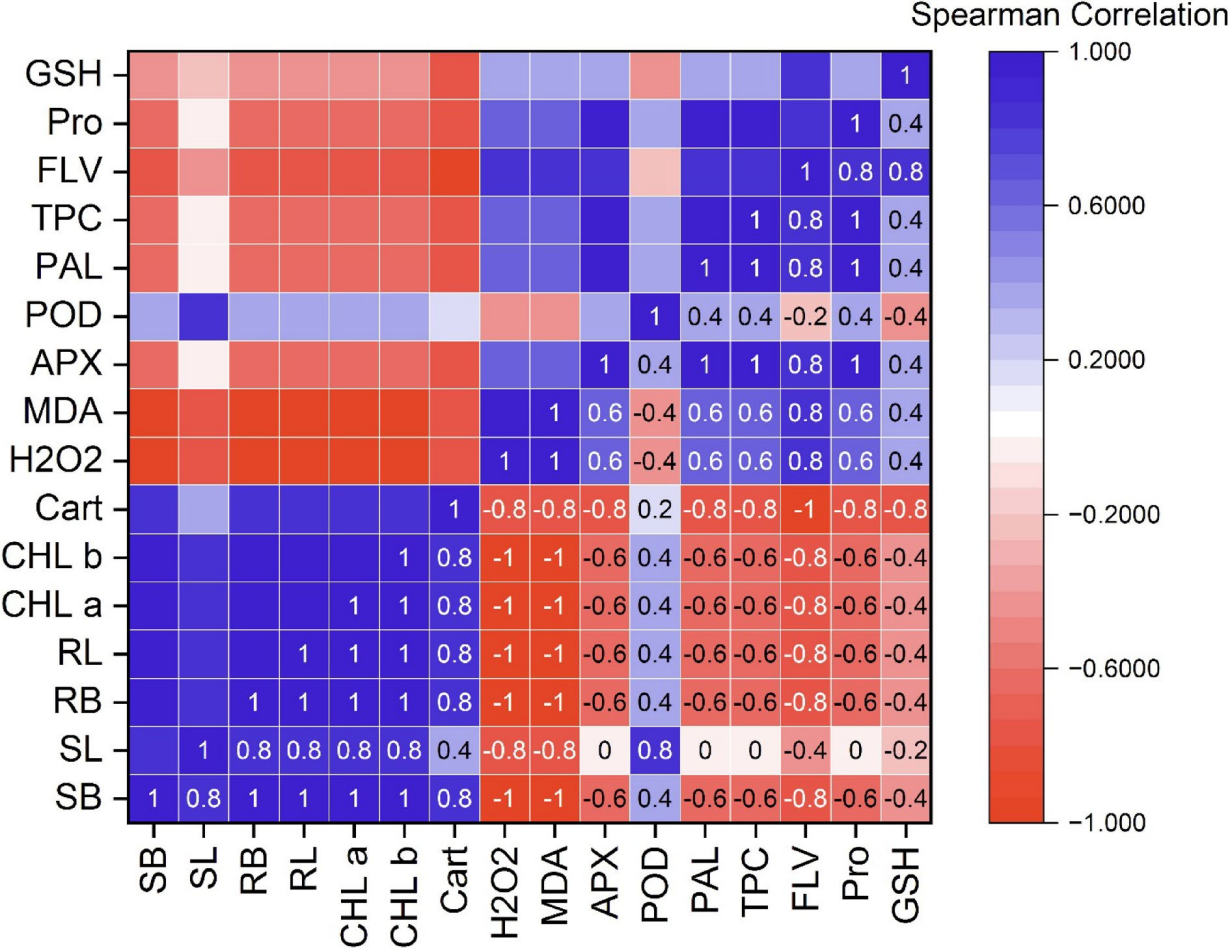
Spearman correlation chart of measured parameters. (SL) shoot length, (SB) shoot fresh biomass, (RL) root length, (RB) root fresh biomass, (Chl a) chlorophyll a, (Chl b) chlorophyll b, (Cart) carotenoid contents, (H_2_O_2_) hydrogen peroxide, (MDA) malondialdehyde, (APX) ascorbate peroxidase, (POX) peroxidase, (PAL) phenylalanine ammonia-lyase activity, (TPC) total phenolic, (FLAV) flavonoids, (Pro) Proline, (GSH) reduced glutathione content.

Photosynthetic pigments are strongly positively correlated with growth parameters, establishing that higher pigment synthesis is associated with enhanced plant growth. Additionally, the analysis reveals a robust positive correlation among chlorophyll-associated parameters, such as chlorophyll a, chlorophyll b, and carotenoids. This means that higher total chlorophyll levels correspond to increased levels of both chlorophylls a and b, along with a higher carotenoid content. Furthermore, antioxidant enzymes show negative correlations with plant growth parameters, indicating that higher antioxidant activity may be related to stress rather than growth (Fig. 8).

## Discussion

Abiotic stress such as soil salinization significantly inhibited the growth and yield of cereal crops and induced oxidative stress. Crops could deal with oxidative injuries generated by salt stress by enhancing their antioxidant defense system. However, if their antioxidant defense is puny, exogenous application of plant biostimulants could help them to increase resistance to abiotic stress condition^31^. Among such biostimulants, microalgae are the one posing antioxidant properties, thus they can increase abiotic stress tolerance. Cyanobacteria (*Spirulina platensis*) has been reported to relieve vegetables under salt stress^12^. Recent studies have further highlighted the biostimulatory effects of *S. platensis* in various crops. For instance, El-Shazoly et al.^32^ demonstrated that SPE application enhanced drought tolerance in Egyptian wheat cultivars, improving growth parameters and physiological responses under water-deficit conditions. Similarly, Elnajar et al.^42^ reported that SPE mitigated drought stress in wheat by enhancing antioxidant enzyme activities and osmoprotectant accumulation. Additionally, Seğmen et al.^33^ found that the application of *Spirulina* extracts significantly improved the yield and quality of pepper, while Elarroussia et al.^34^ observed enhanced growth in tomato and pepper plants. These findings provide further support for the potential of SPE in improving stress tolerance across different plant species. This study investigated the morphological, physiological and biochemical mechanism of *Spirulina* extract (SPE) regulation of wheat seedlings under salt stress.

In this study, the wheat seedlings exposed to salt stress significantly decreased growth attributes (Fig. 1). Salt-stressed seedlings produced less biomass, shoot and root length, and showed phytotoxic symptoms. The negative impacts of salt stress have been formerly recognized in cases of *Sweet Pepper*^35^, *Solanum melongena*^36^, and *Brassica napus*^37^. Similar decreases in plant growth were observed in earlier studies probably due to osmotic, oxidative, and ionic stress^38,40,40^.

Exogenous application of biostimulants is used to promote plant growth and counteract the destructive effects of abiotic stressors^4,41,43,43^. Under salt stress, SPE application remarkably improved seedling growth characteristics (Fig. 1). Previous studies reported that biomass production and growth were significantly inhibited by salt stress in *Solanum lycopersicum*, but the growth attributes were significantly reinforced by water extracts of *Spirulina* application^9–13^. Foliar application of cyanobacteria unusually enhanced the vegetative growth of wheat plants under herbicide exposure^44^. Moreover, exogenous SPE improved the growth of common bean and rosemary under heavy metal stress^13^, and wheat under drought stress condition^45^. These results align with earlier findings on the biostimulatory role of *Spirulina platensis* in improving wheat growth under abiotic stress conditions^46,47^, further validating its efficacy in enhancing crop resilience.

Salt stress imposes oxidative damage on the cells and triggers over-accumulation of reactive oxygen species (ROS), which leads to membrane lipid peroxidation^48^. In the current study, salt stress induced H_2_O_2_ generation and MDA accumulation in wheat seedlings, suggesting that wheat seedlings suffer from oxidative damage (Fig. 3). However, SPE application alleviated these damaging effects of salt stress. In line with our findings, Gharib et al.^11^ reported efficient H_2_O_2_ detoxification in *Spirulina*-treated, cadmium-stressed *Rosmarinus officinalis*. Osman et al.^44^ showed decreased accumulation of MDA in wheat plants under herbicide stress when amended with cyanobacteria extract. The application of SPE successfully alleviated the lipid peroxidation caused by salt stress, which in turn resulted in better growth of seedlings. Studies have shown that *S. platensis* regulates the harmful effects of heavy metals (HMs) and salinity on common bean growth by reducing ROS levels, thereby promoting plant development^13^.

Salt stress has been stated to induce ROS generation in plants, triggering cell protein degradation and membrane damage, thus inspiring oxidative stress^49^. In the current study, the activity of APX increased significantly, but POD activity decreased significantly in salt-affected seedlings compared to the control. Likewise, Touzout^48^, reported that the activities of H_2_O_2_-detoxifying enzymes improved in tomato leaves under salt stress, indicating the effect of salt causing H_2_O_2_ over-generation and consequently stimulating the catalytic activity of APX in tomato seedlings. In this study, foliar application of SPE led to the effective detoxification of H_2_O_2_ during salt stress by up-regulating the activity of APX and POD compared to that in the NaCl-treated seedlings (Fig. 4A). Similarly, previous studies reported that plants antioxidant enzyme activities can be strengthened by applying *Spirulina* as a biostimulant^9,11–13,50^. Thus, the beneficial effect of SPE might improve crop tolerance to salt condition^51,52^. Also, Taha et al.^9^ reported that *Phaseolus vulgaris* plants treated with SPE exhibited higher APX enzyme activity under salt stress compared to non-treated plants, indicating that treated plants experienced lower levels of oxidative damage. Similarly, Sariñana-Aldaco et al.^52^ found that tomato seedlings enhanced their enzymatic antioxidant activity when exposed to salt stress. Overall, SPE foliar application stimulated antioxidant enzyme defense mechanisms, resulting in reduced H_2_O_2_ accumulation, which alleviated membrane injury under salt stress.

Under harsh environmental condition, plants accumulate secondary metabolites, which effectively increase the tolerance of many plants species^53^. Phenolics acids and flavonoids play protective roles as antioxidants, limiting the accumulation of free radicals, stabilizing macromolecules and cellular structures, and supporting cellular redox balance^54^. In this study, a significant increase in total phenolics and flavonoid levels was observed in response to NaCl stress, demonstrating the protective effects of secondary metabolites. Touzout et al.^48^ reported that flavonoids function as antioxidants in the leaves of tomato seedlings under salt stress (150 mM NaCl). However, SPE supplementation significantly enhanced total phenolic content, which was correlated with high antioxidant activity. In the present experiment, total phenolic levels were positively correlated with salt tolerance in wheat seedlings, highlighting the crucial role of phenolics in improving salt tolerance. Interestingly, our results demonstrated that SPE enhanced seedling salt tolerance through the upregulation of PAL activity. PAL is a rate limiting enzyme in the phenylpropanoid pathway^55^, which produces phenols and flavonoids, which is an important antioxidant involved in ROS detoxification in plants^56^. It has been reported that PAL enzymes stimulate the production of secondary metabolites, alleviating oxidative damage and promoting salt stress tolerance^8^. Additionally, in common beans, SPE treatment increased flavonoid content, thereby conferring high antioxidant capacity under HMs stress^13^. Taken together, SPE application stimulates secondary metabolism by enhancing total phenolic and flavonoids accumulation, playing a key role in antioxidant protection against harmful ROS and oxidative damage caused by salt stress.

The GSH content increased significantly under salt stress. Interestingly, it was observed that the co-application of NaCl and SPE restored GSH levels close to those of the control group (Fig. 5D). This suggests that the biostimulant effect of *Spirulina* enhances tolerance to NaCl stress while simultaneously reducing GSH content in SPE-treated wheat seedlings.

Proline, an important osmoprotectant, plays a key role in plant tolerance to abiotic stress^57^. In the present study, salt stress increased proline accumulation in the leaves of wheat seedlings (Fig. 5C), demonstrating a biochemical adaptive mechanism. Similarly, elevated proline levels under salt stress have been observed in wheat^58^ and brinjal^36^. Proline is known to protect against membrane lipid peroxidation and stabilize photosystem II protein pigment complexes under salt stress^59^. Accumulated proline contributes to intercellular osmoregulation (osmotic adjustment) and is closely associated with enhanced stress tolerance^38^. Interestingly, the exogenous application of SPE mitigated the harmful effects of salt stress while increasing proline content in wheat seedlings. Cyanobacteria have also been shown to alleviate salt stress damage in tomato^60^ and *Phaseolus vulgaris*^9^. In stressed seedlings, NaCl triggers complex and interconnected oxidative, ionic, and osmotic responses. Therefore, SPE application could serve as an eco-friendly strategy to protect plants from salt stress by modulating proline accumulation. Increased proline content is crucial for improving tolerance to various abiotic stress conditions^61^.

Although this study demonstrates the beneficial role of *A. platensis* in improving wheat growth under salt stress through physiological and biochemical responses, it does not include gene-level analysis to identify specific molecular mechanisms or stress-responsive pathways involved. The absence of such transcriptomic or gene expression data (e.g., genes related to ion transport, antioxidant defense, or osmotic adjustment) is a limitation that should be addressed in future research. Integrating molecular tools such as qRT-PCR or RNA-seq could provide valuable insights into the regulatory networks activated by *A. platensis* treatment under salinity stress. Moreover, future investigations should incorporate key stress biomarkers such as relative water content, membrane stability indicators (e.g., electrolyte leakage), and the activity of antioxidant enzymes (e.g., SOD, GR, MDHAR, DHAR) to establish a more holistic understanding of the protective mechanisms elicited by *Spirulina*. These additions will help to validate and expand the current findings at the cellular and molecular levels.

Overall, our study revealed another versatile role of SPE as a biostimulant that modulated antioxidant activity and secondary metabolism biosynthesis and played a key role in seedlings salt stress tolerance (Figs. 4C and 5A-B). Application of *S. platensis* showed potential for improving crop tolerance to salt stress. Furthermore, we assumed that *S. platensis* may exhibit major potential in the field of agriculture, particularly in improving the growth and yield of crop plants through increasing their tolerance ability.

## Conclusion

In the current investigation, salt stress significantly inhibited the growth and development of wheat seedlings, including the dropped growth parameters, decreased chlorophyll pigments, destroyed cellular structures, and inhibited antioxidant defense system. Conversely, SPE application improved the antioxidant defense system, subsequently reduced H_2_O_2_ accumulation, and protected membrane integrity. Moreover, we found that the influence of SPE on salt-stressed wheat was double-effect. Firstly, as a main actor in the growth of wheat seedlings, it significantly promoted pigments production, allowing wheat to grow better under salt toxicity. Simultaneously, it dramatically enhanced antioxidative defense mechanism in wheat seedlings probably due to the stimulation of the secondary metabolism enzymes and metabolites which further led to the detoxification of harmful ROS and enhanced growth. Our results showed that spirulina extracts have a high potential for use as a biostimulant in modern agriculture due to their safe nature, ease of use, low cost and ecofriendly approach, but further studies by extracting the active biomolecules contained in the spirulina and determining the biostimulatory effects of each substance on the plants under abiotic stress is required.

## Data Availability

The data that support the findings of this study are available on request from the corresponding authors.
